# Cerebral β-Amyloidosis in Mice Investigated by Ultramicroscopy

**DOI:** 10.1371/journal.pone.0125418

**Published:** 2015-05-27

**Authors:** Nina Jährling, Klaus Becker, Bettina M. Wegenast-Braun, Stefan A. Grathwohl, Mathias Jucker, Hans-Ulrich Dodt

**Affiliations:** 1 Department of Bioelectronics, FKE, Vienna University of Technology, Vienna, Austria; 2 Section of Bioelectronics, Center for Brain Research (MUW), Vienna, Austria; 3 Department of Cellular Neurology, Hertie Institute for Clinical Brain Research, University of Tübingen, Tübingen, Germany; 4 German Center for Neurodegenerative Diseases (DZNE), Tübingen, Germany; Universidad de Sevilla, SPAIN

## Abstract

Alzheimer´s disease (AD) is the most common neurodegenerative disorder. AD neuropathology is characterized by intracellular neurofibrillary tangles and extracellular β-amyloid deposits in the brain. To elucidate the complexity of AD pathogenesis a variety of transgenic mouse models have been generated. An ideal imaging system for monitoring β-amyloid plaque deposition in the brain of these animals should allow 3D-reconstructions of β-amyloid plaques via a single scan of an uncropped brain. Ultramicroscopy makes this possible by replacing mechanical slicing in standard histology by optical sectioning. It allows a time efficient analysis of the amyloid plaque distribution in the entire mouse brain with 3D cellular resolution. We herein labeled β-amyloid deposits in a transgenic mouse model of cerebral β-amyloidosis (APPPS1 transgenic mice) with two intraperitoneal injections of the amyloid-binding fluorescent dye methoxy-X04. Upon postmortem analysis the total number of β-amyloid plaques, the β-amyloid load (volume percent) and the amyloid plaque size distributions were measured in the frontal cortex of two age groups (2.5 versus 7-8.5 month old mice). Applying ultramicroscopy we found in a proof-of-principle study that the number of β-amyloid plaques increases with age. In our experiments we further observed an increase of large plaques in the older age group of mice. We demonstrate that ultramicroscopy is a fast, and accurate analysis technique for studying β-amyloid lesions in transgenic mice allowing the 3D staging of β-amyloid plaque development. This in turn is the basis to study neural network degeneration upon cerebral β-amyloidosis and to assess Aβ -targeting therapeutics.

## Introduction

Alzheimer´s disease (AD) is a progressive neurodegenerative disorder, which is characterized by a variety of neuropathological abnormalities [1]. One pathological hallmark of AD is the extracellular deposition of aggregated amyloid-beta peptide (A β) in the brain parenchyma (β-amyloid plaques). A variety of mouse models have been generated that recapitulate the amyloid aspect of the disease [2,3]. Today these models play a key role in developing Aβ-amyloid targeting therapeutics and diagnostics.

For visualizing β-amyloidosis in transgenic mouse brains, imaging techniques such as positron emission tomography (PET) [4,5], 2-photon imaging [6,7] and confocal imaging (e.g. [8]) can be applied. PET imaging, where amyloid deposits can be labeled by e.g. the Pittsburgh Compound B (PIB; [9]), has the advantage to allow observations of entire mouse brains over time. However, its resolution is rather low. Multi- or 2-photon imaging enables the visualization of β-amyloidosis via labeling with e.g. methoxy-X04 [10] over time in combination with high spatial resolution. Nevertheless, here the observation field and the penetration depth are too limited to allow the analysis of amyloid plaque distribution in the entire mouse brain [11]. To obtain high resolution imaging of β-amyloid plaques in the entire brain, a possible alternative approach could combine confocal laser scanning microscopy with serial section reconstruction of Aβ-stained brain slices. However, obtaining an overview of β-amyloid distributions in the entire brain by serial sectioning of cm-sized tissues is difficult and time consuming.

Therefore a technique is required, which allows visualization of β-amyloid plaques in high resolution in the non-dissected brain. Here, ultramicroscopy (light sheet microscopy) [12] closes a gap and provides a new way of pathological analysis of AD transgenic mice. In ultramicroscopy (UM) mechanical slicing is replaced by optical sectioning. The obtained fields of view are in the centimeter range. Thus, using UM in combination with the amyloid-specific dye methoxy-X04 allows the analysis of all β-amyloid plaques in the entire mouse brain.

Since the resolution of the applied UM-setup, equipped with a 4x Olympus Fluor objective of N.A. 0.28 provides a lateral resolution < 2 μm and a resolution of about 5 μm along the detection pathway, even small plaques can be directly counted and reconstructed in 3D. Since the image resolution slightly degrades towards the edges of the field of view, we started plaque counting at 8 μm diameter to be on the safe side.

In this study, we utilized UM to analyze β-amyloid deposition in the APPPS1 mouse model of cerebral β-amyloidosis [13]. This mouse line shows a significant increase of Aβ-deposits between 1 and 8 months of age [13]. In the neocortex of these mice the first amyloid plaques can be detected at the age of 4–6 weeks. Here, we analyzed APPPS1 mice of two groups quantitatively, which were divided in a young group (2.5 months) and an adult group (7–8.5 months), comprising 5 animals each. This approach allowed us to quantify β-amyloid plaque loads (volume %), total β-amyloid plaque numbers and the β-amyloid plaque size distributions with respect to the different age groups.

The aim of this proof-of-principle study was to analyze the formation of β-amyloid plaques in mouse brains with a fast and user-friendly 3D-imaging technique. UM allows a time efficient 3D-examination and direct evaluation of the β-amyloid load. Thus it can be applied for characterizing disease stages in the mouse brain. This in turn is an important basis to access neural network changes upon cerebral β-amyloidosis and Aβ-targeting therapeutics.

## Material and Methods

### Mouse model

In this study APPPS1 mice were analyzed as a model for cerebral amyloidosis via UM. APPPS1 mice co-express human APP carrying the K670N/M671L “Swedish” double mutation and human PS1 L166P under the control of the neuron specific THY1 promoter and were generated on a C57BL/6J genetic background [13]. Due to the aggressive PS1 mutation, which results in a very high Aβ42 to Aβ40 ratio, APPPS1 mice show a rapid onset of cerebral β-amyloidosis before 2 months of age.

### Methoxy-X04-labeling and chemical clearing

We herein examined the brains of APPPS1 transgenic mice at the age of 2.1–2.7 (n = 5) and 7–8.5 (n = 5) months of age. All animal care and euthanasia was done in accordance and approved by the veterinary office regulations of Baden-Württemberg (Regierungspräsidium Tübingen, Germany). Sacrificing of the animals was done in the Hertie Institute for Clinical Research, 72076 Tübingen, Germany by the authors of this study using Ketamin/ Xylazin and transcardial perfusion. Raising and breeding of mice was done at the animal care facilities of the Hertie Institute for Clinical Research, 72076 Tübingen, Germany. Approval was obtained by an ethics committee of the Regierungspräsident Tübingen (approval number N12/07). All efforts were made to minimize suffering. Mice were injected twice (24 h interval) intraperitonially (i.p.) with 75 μl of 10 mg/ml methoxy-X04 (courtesy of Dr. WE Klunk) in dimethyl sulfoxide. Two hours after the last injection animals were deeply anesthetized (400 mg/kg ketamine, 40 mg/kg xylazine) and transcardially perfused with phosphate-buffered saline (PBS) at 4°C, followed by 4% paraformaldehyde in PBS. Brains were prepared and postfixed in 4% paraformaldehyde in PBS at 4°C overnight, followed by three washes in PBS. The tissue was dehydrated in an ascending series of ethanol concentrations (50%, 70%, 80%, 96% for 24h, 4x 100% for 24h) and transferred into the peroxide free clearing medium, containing 1 part benzyl alcohol and 2 parts benzyl benzoate (BABB), [14]. The brains were kept in this clearing solution until they became almost transparent.

### Ultramicroscopy and 3D-reconstruction

**Fig 1:**
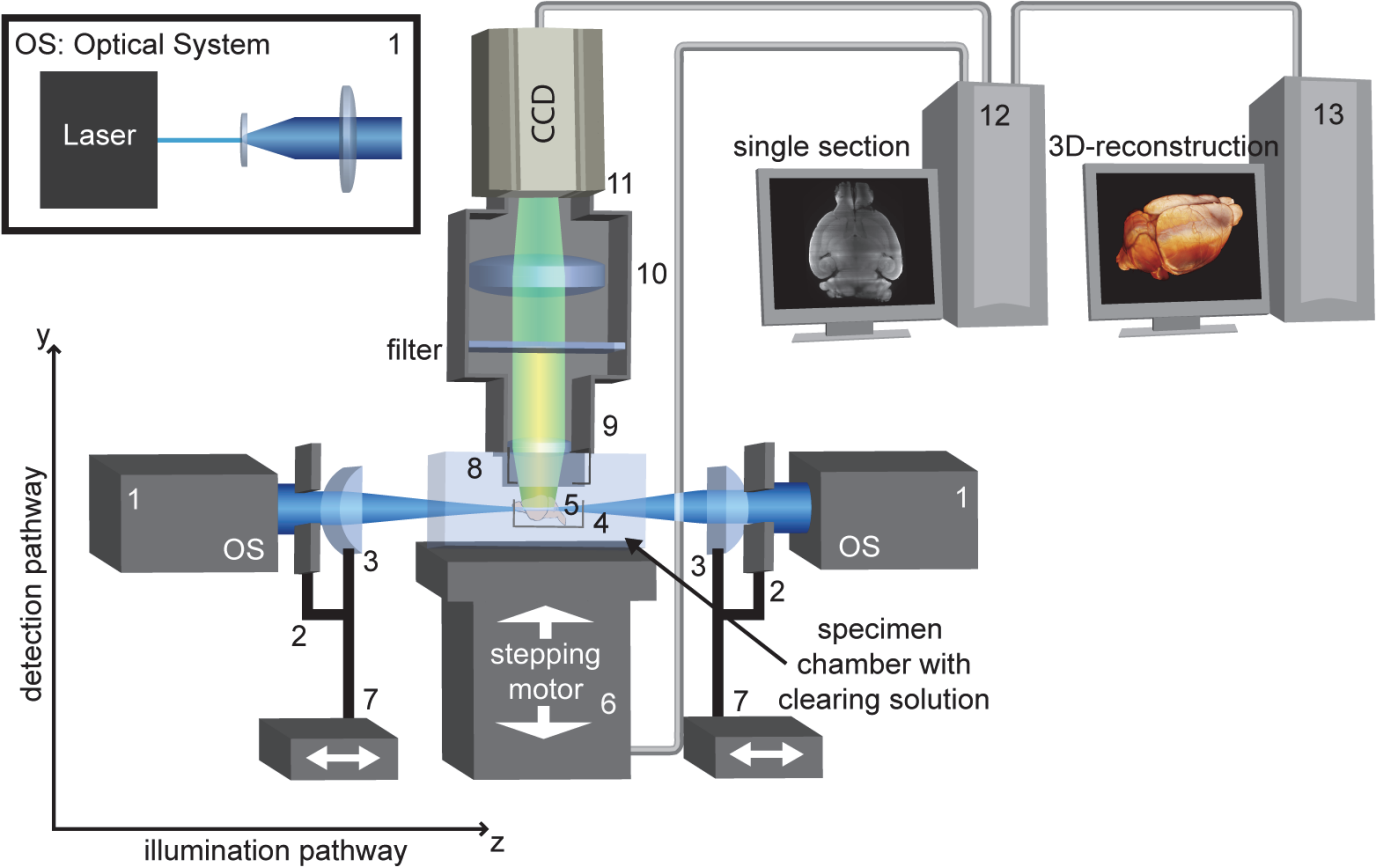
Ultramicroscopy setup. Principle of standard UM consisting of a slit aperture and a single cylindrical lens on each illumination side. The transparent brain is illuminated perpendicular to the observation pathway by a laser forming a thin sheet of light. The emitted fluorescence light is projected to a camera target by an objective, while a matched optical filter blocks the excitation light. By moving the specimen through the light sheet a stack of images is recorded. From these data a 3D-reconstruction is calculated by software. 1) Optical system (OS), which generates a magnified parallel beam. This beam is divided into two equal parts using a beam splitter. 2) Rectangular-slit aperture, 3) cylindrical lens, 4) clamp, 5) brain, 6) Sy: computer-controlled elevation (y-direction), 7) Sz: computer-controlled linear stage (z-direction), 8) immersion cap, 9) objective, 10) tube lens(es), 11) camera target, 12) computer with imaging software, 13) computer with 3D reconstruction software.

For ultramicroscopy, we used the standard setup described [15] (Fig 1). Fluorescence excitation of the methoxy-X04 labeled amyloid plaques was done with a 405 nm, 180 mW diode laser (Newport, CA, USA). The excitation light was blocked by an optical lowpass filter with a cutoff of 455 nm (AF-Analysentechnik, Germany). Image recording was performed using Olympus XL Fluor objectives (2x: N.A. 0.14; 4x: N.A. 0.28) and a 12 bit CCD camera with 2048 * 2048 pixels resolution (Cool Snap K4, Roper Scientific, Germany). For visualizing the entire brain a 0.63 x post-demagnification was combined with the 2x-objective. 3D image reconstruction was performed via Amira 5.2 (Visage Imaging Germany). For image processing a computer system with two Opteron Dual Core processors and an NVIDIA Quadro FX5800 graphic processor board with 4 GB frame buffer size (NVIDIA, USA) was utilized.

### Image segmentation

3D-amyloid plaque segmentation data were obtained by applying the semi-automatic threshold algorithm module “Segmentation Editor” in Amira 5.2. Segmentation of the methoxy-X04 labeled amyloid plaques was technically feasible, as they appear with high contrast. Under manual control, areas of blood vessels, which occur due to incomplete perfusion, and rare observed fine stripes, apparent as artifacts of UM methods, were excluded from segmentation.

Further image segmentation procedures and statistics are described in the result section; statistical analysis was performed with Sigma Plot (Systat Software Inc., Chicago, USA). Statistically significances are indicated by asterisks (* *p* < 0.05, ** *p* < 0.01, *** *p* < 0.001).

## Results

**Fig 2:**
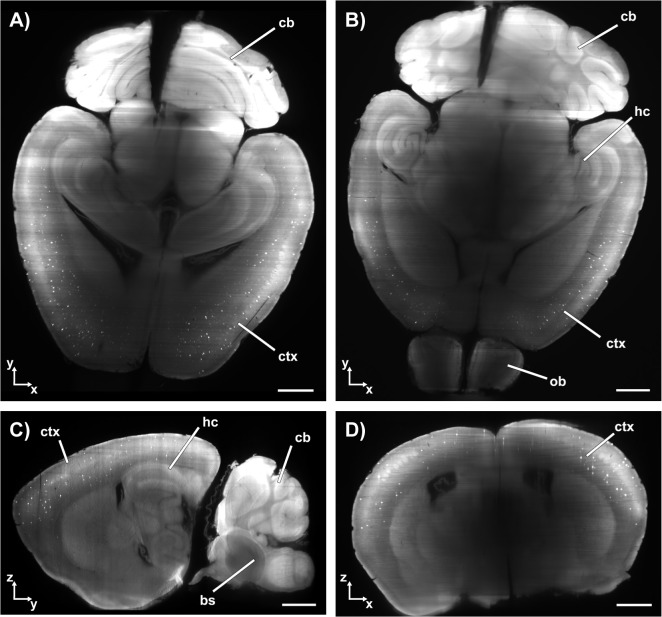
UM´s example images of APPPS1. Cross sections in the orthogonal directions are used to analyze the β-amyloid plaque distribution in the entire mouse brain of APPPS1. The β-amyloid plaques appear as bright dots in the neocortex (example of an animal of the young group is shown). A) Transversal plane (x,y); B) transversal plane (x,y); C) Computed sagittal plane (yz); D) computed coronal plane (xz). ctx: cortex; cb: cerebellum; hc: hippocampus, bs: brainstem; ob: olfactory bulb.

Via visual analysis of the single images of each stack, the β-amyloid plaque distribution was examined for each APPPS1 mouse brain. Fig 2 shows how the β-amyloid plaque distribution was recorded two dimensionally by optical sectioning. The young group shows modest β-amyloid deposition in the cortex and minor deposition in the hippocampal region. In contrast, robust β-amyloid deposition throughout almost the entire brain is observed in the adult mice. In accordance with earlier results [13], β-amyloid deposition in the cerebellum was less compared to other brain regions. It should be noted that structures deep within the brains of the adult animals were difficult to analyze, as these brains were not completely transparent.

**Fig 3:**
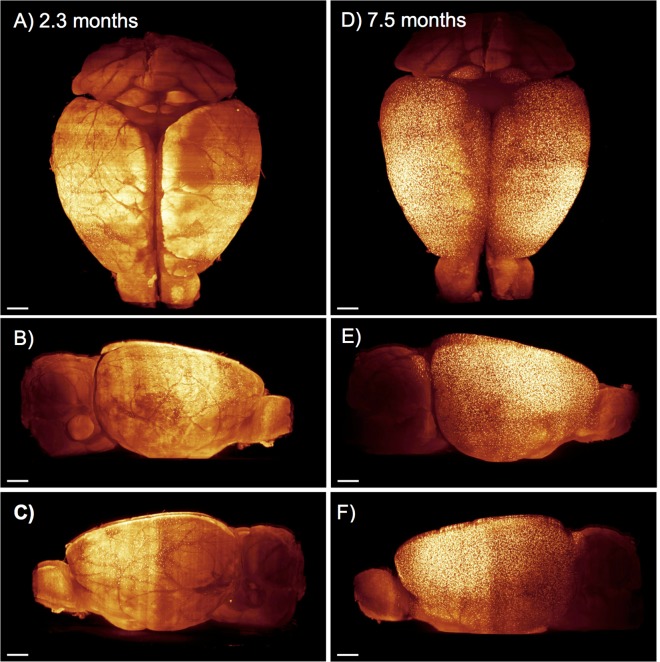
Comparison between a young (2.3-month-old) and adult (7.5-month-old) APPPS1 tg brain. 3D reconstructions demonstrate an overall age-related increase in β-amyloid plaques. The maximum intensity projections of reconstructed images show a higher plaque density with increasing age. Brain from a mouse at 2.3 months of age viewed from the top (A), from the right side (B) and left side of (C). D) Brain from a mouse at 7.5 months of age viewed from the top (D), from the right side (E) of and left side (F). Scale bar: 1mm.

UM allows a straightforward and fast 3D analysis of the entire mouse brain. Fig 3 shows 3D brain reconstructions of two representative animals from the young and the adult group. As expected the β-amyloid plaque density in the entire brain is much higher in the adult than in the young group.

**Fig 4:**
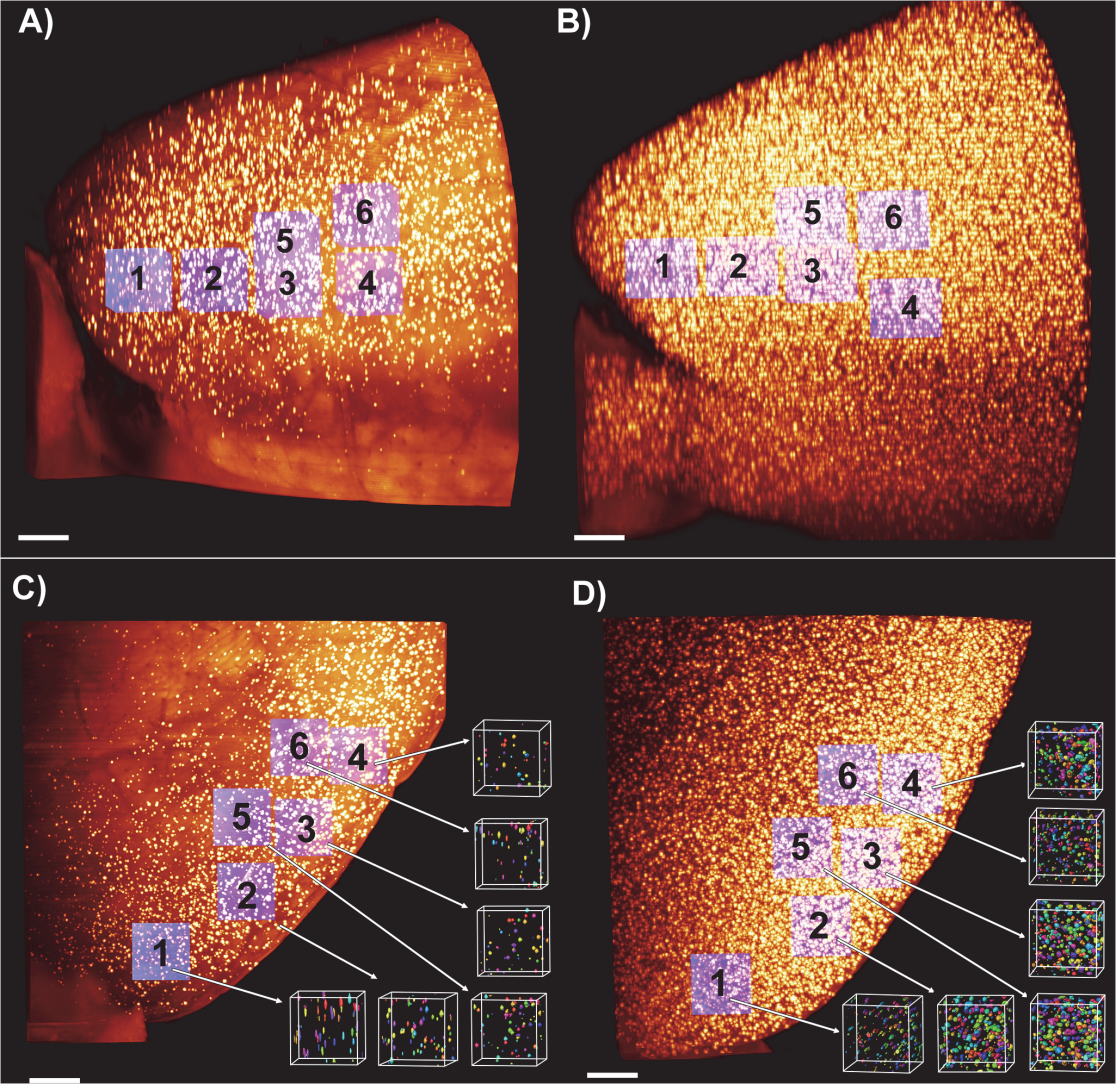
Location of measured test-cubes. β-Amyloid plaques (yellow dots) in the right hemisphere of the frontal cortex in the APPPS1 mouse model, side view: example from the young (2.7 month-old) group (A) and the adult (7.8 month-old) group (B). Positioning of the six cubed-shaped areas (purple color) in the frontal cortex for measuring the β-amyloid plaque volumes by applying a threshold segmentation technique. C-D) Top view of the frontal cortex: example from the young group (C) and old group (D). After segmentation amyloid plaque volumes of the six cubed shaped areas are represented in various colors. Scale bar 500 μm.

The β-amyloidosis in the frontal cortex of the brains was quantitatively analyzed. The brain volumes of individual animals vary. Hence, the volume of each cortex was determined by segmentation (raw data based on data recorded with the 2-fold objective). There was no significant difference in the cortex volume between the age groups. The total β-amyloid plaque number and the volume of amyloid plaques was measured within cubed shaped blocks placed at different regions of the frontal cortex (raw data based on data recorded with the 4-fold objective). Fig 4 shows how six of these cubes were positioned in the frontal cortex in each brain hemisphere. To adjust for potential differences due to tissue processing, the volume of each test cube was fixed to 0.1% of the determined cortex volume. There was no significant difference in the cube shaped block volume between the age groups (S1 Table). Labeled amyloid plaques were segmented based on intensity, using a manual threshold on Amira 5.2 visualization software.

**Fig 5:**
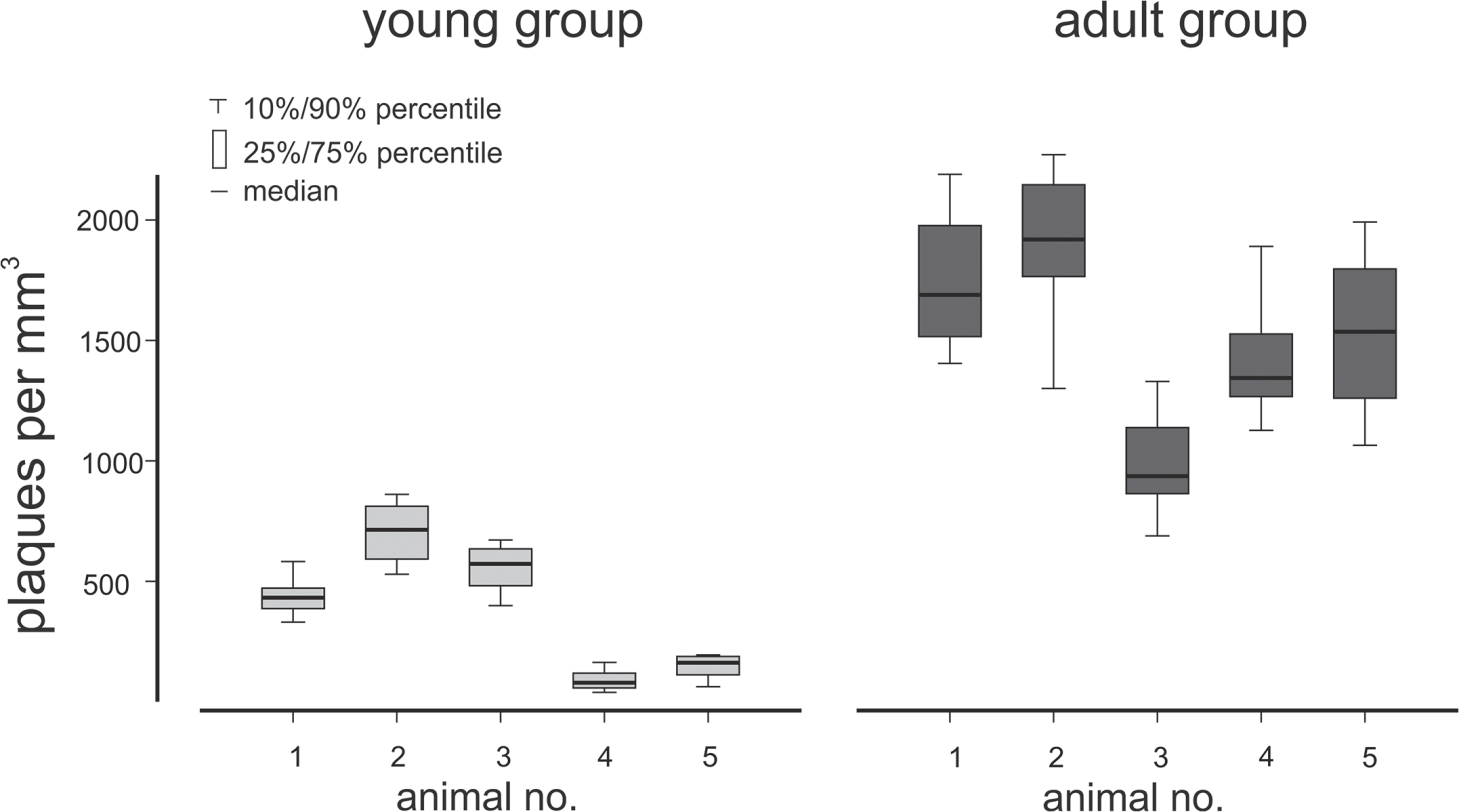
Quantification of the number of amyloid plaques per mm^3^. The total β-amyloid plaque number per mm^**3**^ was obtained from six sample cubes, acquired within the frontal cortex of young (2.5 months) and adult (7–8.5 months) APPPS1 tg mice. The total number of plaque per mm^**3**^ increases with age.

Fig 5 shows that the total plaque number, i.e. β-amyloid plaques per sample cube of each animal, is increased in the adult group. In all sample cubes of the young group together 4411 β-amyloid plaques were counted, whereas in all samples cubes of the adult group together 17829 β-amyloid plaques were counted, i.e. the β-amyloid plaque number has quadrupled with age. Counting of plaques was done by the particle segmentation algorithm implemented in Amira 5.2. Within both age groups, there was a high variability, i.e. there were significant differences in the total plaque numbers of animals within one group (p<0.001, Kruskal-Wallis on Ranks) (Fig 5). However, there is no obvious relationship between the age of the individual animals within one group and the plaque numbers (S1 Fig).

**Fig 6:**
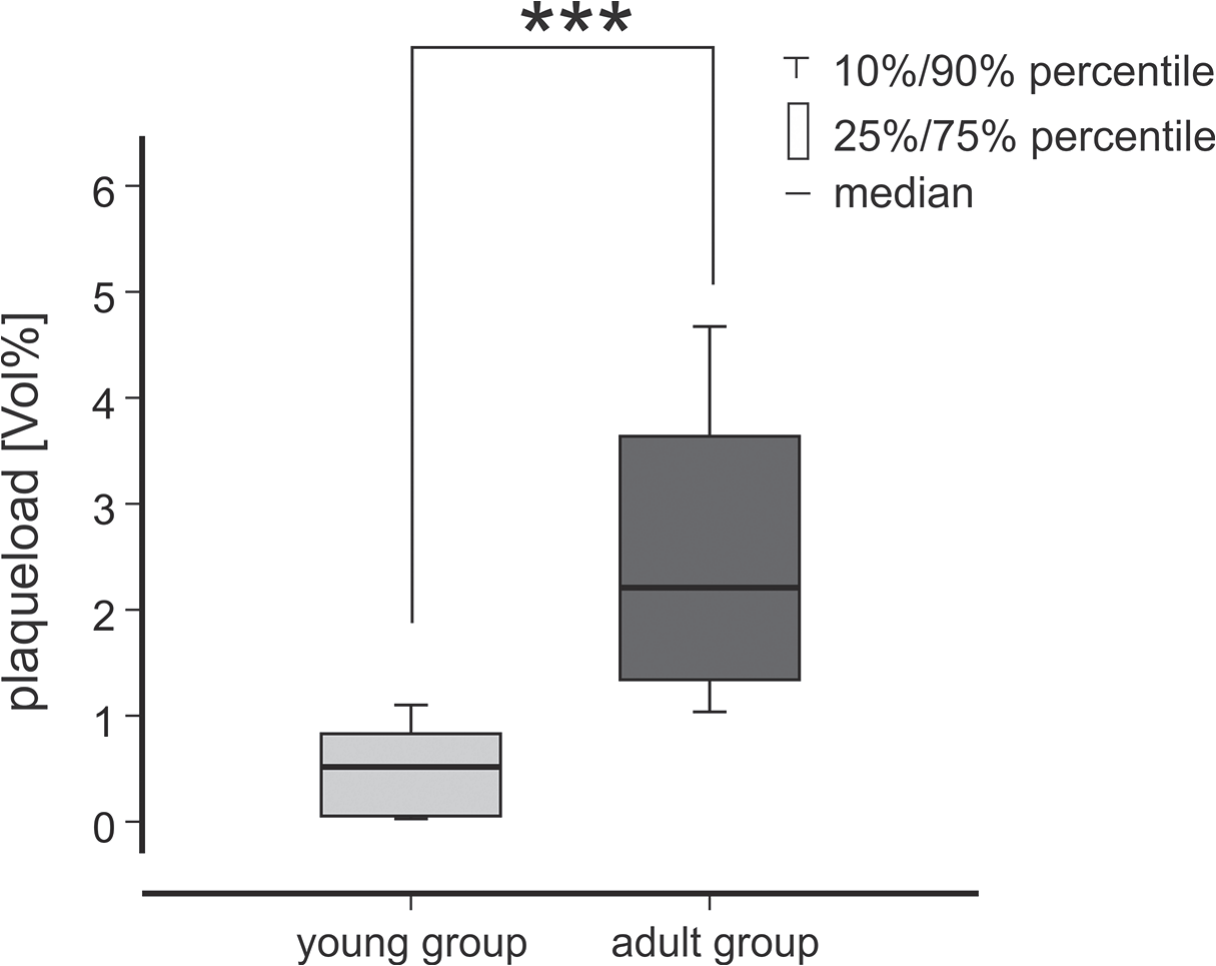
Quantification of the 3D β-amyloid plaque load. The β-amyloid plaque load (volume %) was obtained from six sample cubes, acquired within the frontal cortex of young (2.5 months) and adult (7–8.5 months) APPPS1 tg mice. The β-amyloid plaque load in the adult group is significantly higher compared to the young group (p<0.001, t-test).

Next, we wanted to analyze alterations in the β-amyloid plaque load to compare age-dependent changes in UM and classic immunohistochemistry. Fig 6 depicts that the 3D plaque load, i.e. the volume fraction of the β-amyloid plaques per sample cube, is significantly higher in the adult group than in the young group (p<0.001, Mann-Whitney). This result is in line with stereological results, which were acquired in 2D [13].

**Fig 7:**
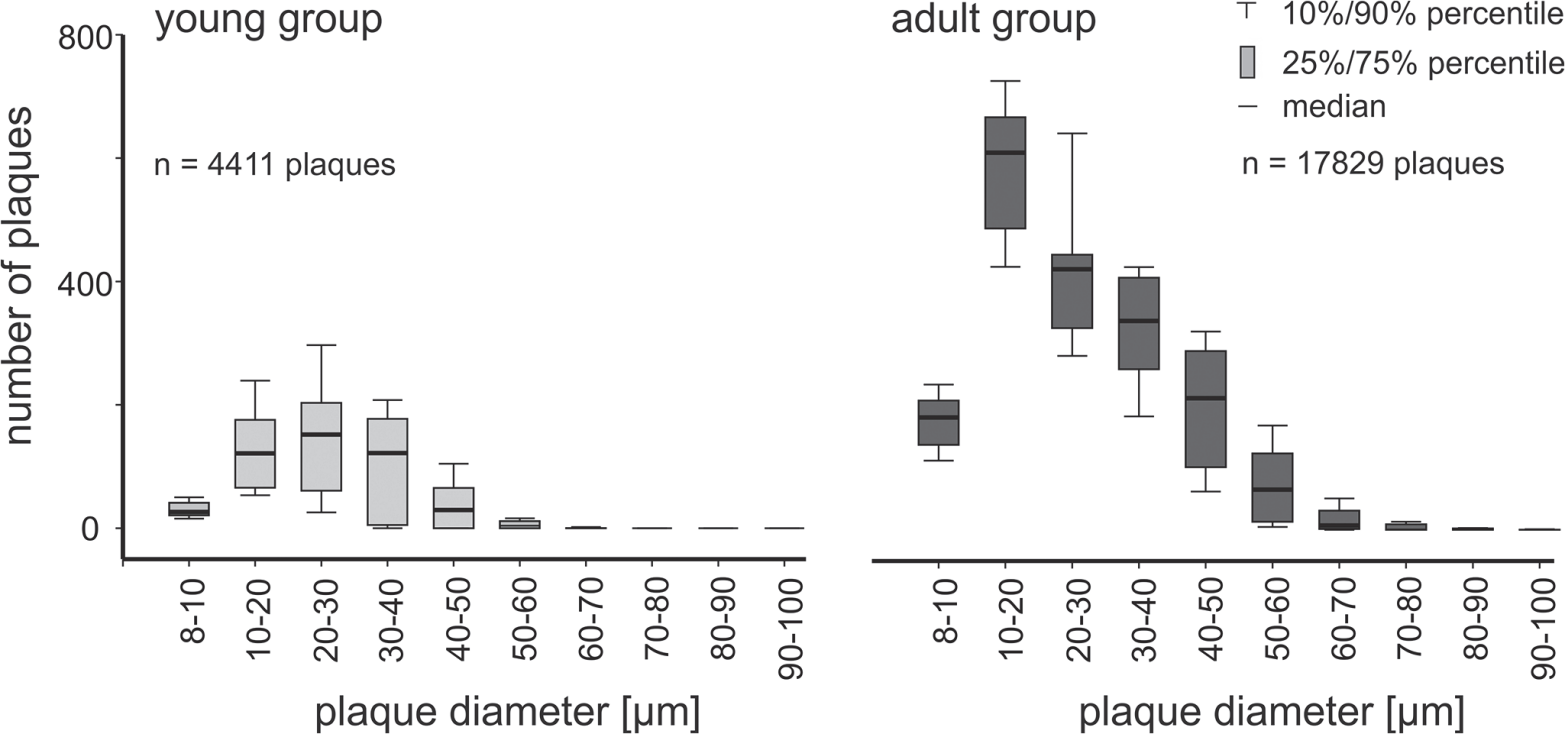
Histograms of plaque diameters. The plaque sizes were binned into size groups of 10 μm for the young and the adult mice. In total, the number of β-amyloid plaques in all size classes was increased in the adult group. All plaques within the young group and the adult group, respectively were counted for histogram generation.

**Fig 8:**
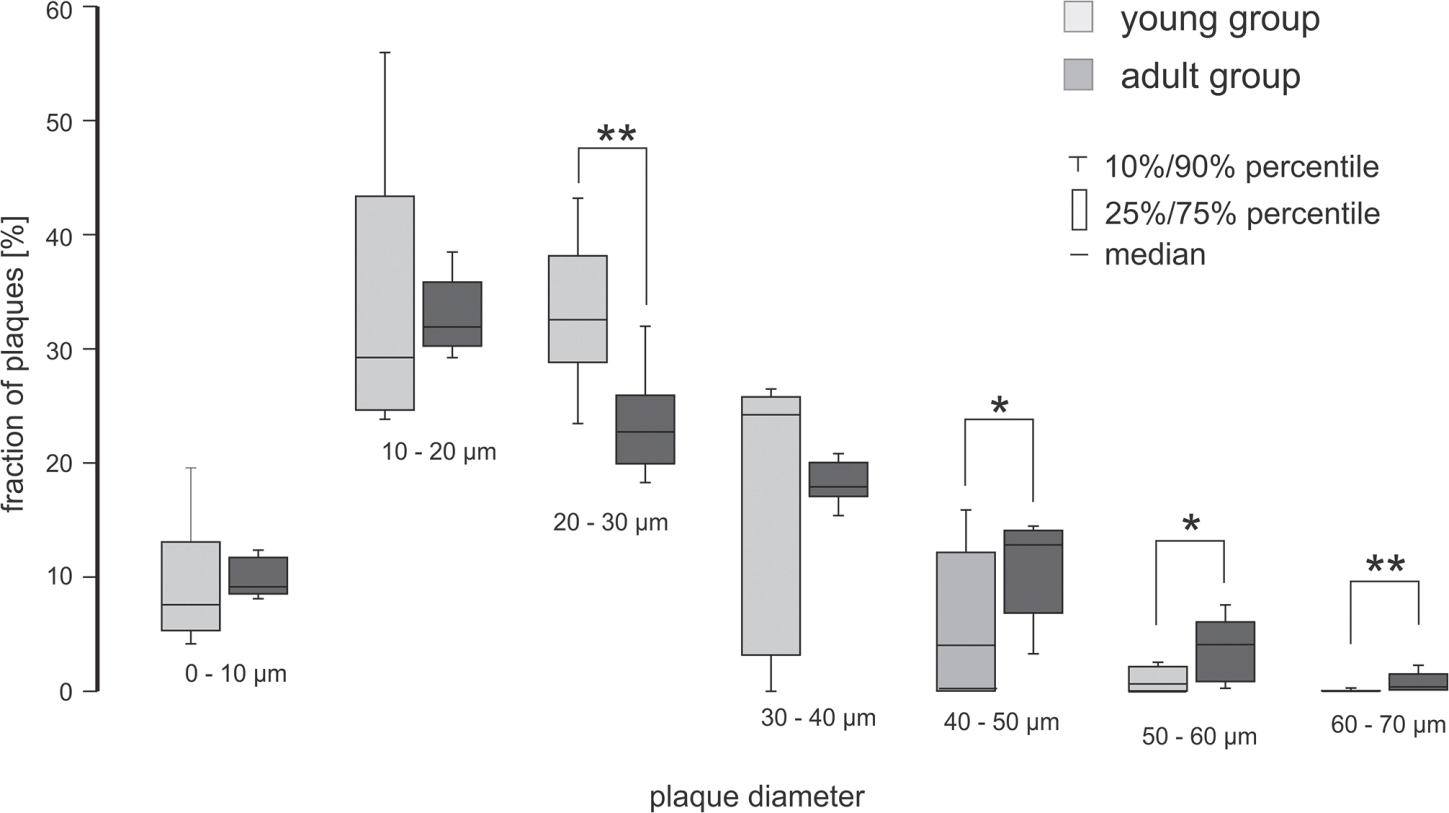
Variations in the fraction of plaques for different diameters. In the β-amyloid plaque diameter categories 40–50μm plaques were relatively more frequent in the adult animals. However, in the diameter group of 20–30 μm plaques were relatively more frequent in the young animals. For the β-amyloid plaque diameter categories 0–20 μm and 30–40 μm no significant differences could be shown. It cannot by excluded that plaques > 50 μm may be due to agglomeration of smaller plaques or artefacts.

Next, we were interested in differences of individual plaque sizes. Therefore, the β-amyloid plaque volumes were assumed to be approximately spherical to provide a more concrete measure for comparison. The conversion from plaque volumes *V* to diameter *d* was done according to the equation $\;d\;=\;2*\sqrt[{3}]{\frac{3V}{4\pi }}$. Fig 7 shows the summarized histograms of the β-amyloid plaque size distribution for the young group and the adult group. In total, the number of β-amyloid plaques in all histogram intervals was increased in the adult group. Fig 8 depicts the relative β-amyloid plaque fraction of the young group compared to the adult group: In the β-amyloid plaque diameter categories 40–70μm plaques were relatively more frequent in the adult group. However plaques with diameters > 50 μm may be due to artefacts caused by limitations in the microscope optics (see discussion). In the size group of 20–30 μm plaques were relatively more frequent in the young animals. For the β-amyloid plaque diameter categories 0–20 μm and 30–40 μm no significant differences could be observed. Therefore, it can be speculated that the fraction of large plaque diameters increase with age.

## Discussion

UM allows a time efficient analysis of the β-amyloid plaque distribution in the entire mouse brain. Orthogonal 2D-slices can be quickly computed in axial, frontal or sagittal direction. Also non-orthogonal slices can be achieved in any arbitrary direction and allow to obtain a detailed overview of β-amyloid plaque distributions in different brain regions. The definition of sample cubes within a brain hemisphere and the counting of all plaques inside this volume can be performed largely automatically. Routinely applied, this procedure should not require more than ~30 min for each brain. Hence the most time consuming part of the method is the chemical tissue clearing procedure, which requires about eight days. However in this step multiple brains can be processed in parallel. In contrast, mechanical slicing of multiple brains usually has to be performed sequentially.

Applying UM imaging we were able to provide evidence that the number of plaques significantly increases with age. Furthermore, we observed a decrease in the percentage of small sized β-amyloid plaques (20–30 μm) in the older group, together with an increase in the percentage of large plaques (40–50 μm), indicating age-dependent plaque growth. This is in line with the findings by [7], where β-amyloid deposits were shown to increase constantly in size over a 6 months imaging period in APPPS1 tg mice, together with a striking decrease in the formation of newly developed plaques at later time points. A conservative interpretation of our results indicates that existing β-amyloid plaques were growing and new plaques were less formed in older animals as observed in [7].

Differently to a previous study using the same mouse model performed by [7], we found a small fraction of plaques (about 5%) having diameters larger than 50 μm. It may be speculated that these plaques are formed by aggregation of small plaques developing in a close neighborhood. They may be also an artefact caused by a diminished axial resolution of our microscopy setup at the edges of the microscopic viewing field, where the thickness of the light sheet rapidly increases due to its limited Raleigh range. This limitation may be markedly diminished by using more advanced future light sheet generator optics [16].

Considering exclusively plaque diameters up to 40 μm the results obtained from our study are well comparable to [7] as demonstrated by Table 1.

**Table 1:** Determined Plaque diameters compared to a previous study.

This study (7–8.5 month old animals)		Study form Hefendehl et al. (7 month old animals)	
Plaque diameter (μm)	Relative percentage	Plaque diameter (μm)	Relative percentage
8–10	11.7%	8–12	12.5%
10–20	38.7%	12–20	36.1%
20–30	27.7%	20–32	37.5%
30–40	21.9%	32–44	13.9%

For comparing these results it has also to be taken into account that the age groups were slightly different (7–8.5 month vs. 7 month in [7]). Due to tissue shrinkage during the clearing process the plaque sizes may be biased by about 20% in direction to lower values [17] However, considering all differences in the experimental approach (in vivo vs in vitro, different lasers, tissue clearing, different imaging systems) our results are comparable to [7].

Due to their outer shape a previous study [13], distinguished three types of β-amyloid plaques in APPPS1 mice via immunohistochemistry. Applied after UM, immunohistochemistry may be a promising way for histological characterization of different plaque types in future studies.

Postmortem analysis such as immunohistochemical staining approaches allows a characterization of the β-amyloid plaques types in 2D. Principally, artifacts from 2D mechanical slicing approaches are not avoidable. Since in UM slicing is replaced by optical sectioning it enables a 3D analysis of cerebral β-amyloidosis and measuring plaque volumes in high resolution.

We demonstrated that UM allows a direct quantification of β-amyloid plaques, herein performed in six sample cubes in the frontal cortex of five mice. One additional mouse of the young group was excluded from this study since it developed nearly no β-amyloid plaques.

The volume of individual β-amyloid plaques was measured within sample cubes by applying a manual threshold segmentation technique. Automatic segmentation approaches were found to be difficult due to variations in signal-to-background ratio, which is due to an inevitable loss of light intensity with increasing imaging depth. This drawback may be overcome by improved tissue clearing techniques such as THF/DBE clearing [17,18], or “CLARITY” [19]. Improved tissue transparency and progress in computational processing could make UM powerful enough to quickly and automatically analyze all β-amyloid plaques of a mouse brain. It provides a fast and powerful alternative to indirect established quantification methods such as stereology.

We demonstrated that virtually the entire amyloidosis in the cerebral cortex can be imaged and segmented using light sheet microscopy. However, it was not possible to visualize plaques in all subcortical regions in all brains. Beside the limited transparency of the brains in older animals, imaging in deeper regions of the brain was hampered by the used objectives, which were not matched to the refractive index matching solution of n = 1.559 (BABB). This drawback can be overcome by using refractive matched objectives of long working distance, which will presumable be made commercially available in the near future.

For the first time, this study provides an evaluated approach to combine 405 nm laser excitation and UM to measure cerebralβ-amyloidosis. We showed that near UV-light passes the clearing medium in the specimen chamber without major refraction or absorption. In previous UM studies, fluorescence generated by GFP or immunostainings was applied for signal detection. Here, we demonstrate that the fluorescent dye methoxy-X04 is sufficiently stable to undergo the dehydration and clearing procedure. The application of other structure-associated or cellular markers, which can pass the blood-brain barrier, might also enable the investigation of other neuronal processes or disease-dependent modifications.

In summary, we have employed the UM imaging technique to generate three-dimensional reconstructions of entire APPPS1 mouse brains by presenting β-amyloid plaque distributions at two different ages. Due to its time efficiency, UM provides a powerful technique to examine disease patterns in entire brains of transgenic mouse models.

## Supporting Information

S1 Fig Relation of plaque numbers and age within each group. (DOCX)

S1 Movie APPPS1 mouse brain with labeled amyloid plaques. Clipping plane, the outline of the brain is surfaced rendered red, while the inner parts are colored in black and white.
(MP4)

S2 Movie Six sample cubes in the frontal cortex (representative of an animal of the adult group is displayed). (MP4)

S3 Movie Animation of a representative animal from the young group (2.3 month-old) is shown with maximum intensity projections of reconstructed images. (MP4)

S4 Movie Animation of a representative animal from the adult group (7.5 month-old) is shown with maximum intensity projections of reconstructed images. (MP4)

S1 Table Volume of sample cubes. (DOCX)
