# Supplementary material for: Cerebral β-Amyloidosis in Mice Investigated by Ultramicroscopy
Source: PLoS One. 2015 May 27;10(5):e0125418. doi: 10.1371/journal.pone.0125418 (PMC4446269; doi:10.1371/journal.pone.0125418)
Supplement: S1 Fig — (DOCX) [file pone.0125418.s001.docx]

**Figure S6. Relation between age and plaque numbers of mice in the young and adult group.** There is no clear linear relationship between age and plaque numbers of mice within one group (young group: r^2^ = 0.77, adult group r^2^ = 0.02).
