# Supplementary material for: Cerebral β-Amyloidosis in Mice Investigated by Ultramicroscopy
Source: PLoS One. 2015 May 27;10(5):e0125418. doi: 10.1371/journal.pone.0125418 (PMC4446269; doi:10.1371/journal.pone.0125418)
Supplement: S1 Table — (DOCX) [file pone.0125418.s006.docx]

Volumes of the six sample cubes per hemisphere:

| **brain No.** | **age** | **Volume (mm^3^)** |
| --- | --- | --- |
| **young group** | | |
| 1 | 2.7 | 0.183 |
| 2 | 2.7 | 0.187 |
| 3 | 2.7 | 0.193 |
| 4 | 2.3 | 0.197 |
| 5 | 2.5 | 0.200 |
| **adult group** | | |
| 1 | 7.8 | 0.188 |
| 2 | 7.1 | 0.197 |
| 3 | 7.5 | 0.196 |
| 4 | 7.5 | 0.207 |
| 5 | 8.5 | 0.194 |
